# The Construction of Common and Specific Significance Subnetworks of Alzheimer's Disease from Multiple Brain Regions

**DOI:** 10.1155/2015/394260

**Published:** 2015-03-19

**Authors:** Wei Kong, Xiaoyang Mou, Na Zhang, Weiming Zeng, Shasha Li, Yang Yang

**Affiliations:** ^1^Information Engineering College, Shanghai Maritime University, Shanghai 201306, China; ^2^DNJ Pharma and Rowan University, Glassboro, NJ 08028, USA; ^3^Psychology Department, The Second People's Hospital of Guizhou Province, Guiyang 550004, China; ^4^Department of Computer Science and Engineering, Shanghai Jiao Tong University, Shanghai 200240, China

## Abstract

Alzheimer's disease (AD) is a progressively and fatally neurodegenerative disorder and leads to irreversibly cognitive and memorial damage in different brain regions. The identification and analysis of the dysregulated pathways and subnetworks among affected brain regions will provide deep insights for the pathogenetic mechanism of AD. In this paper, commonly and specifically significant subnetworks were identified from six AD brain regions. Protein-protein interaction (PPI) data were integrated to add molecular biological information to construct the functional modules of six AD brain regions by Heinz algorithm. Then, the simulated annealing algorithm based on edge weight is applied to predicting and optimizing the maximal scoring networks for common and specific genes, respectively, which can remove the weak interactions and add the prediction of strong interactions to increase the accuracy of the networks. The identified common subnetworks showed that inflammation of the brain nerves is one of the critical factors of AD and calcium imbalance may be a link among several causative factors in AD pathogenesis. In addition, the extracted specific subnetworks for each brain region revealed many biologically functional mechanisms to understand AD pathogenesis.

## 1. Introduction

Alzheimer's disease (AD) is a complex progressive and irreversible neurodegenerative disease. The characteristic pathology change in AD is the deposition of beta-amyloid (A*β*) and poly-Tau protein in the cell. The pathomorphism features of AD are the senile plaques (SP) and neurofibrillary tangles (NFT), cerebrovascular amyloid, dystrophic neuritis, and loss of synaptic connections [1, 2]. AD is a complex neurodegenerative disorder with largely unknown genetic mechanisms. Lots of transcriptome studies show that AD can lead to the dysfunction of multiple brain regions and progressively destroys remembering, thinking, and reasoning skills [3]. Identifying altered gene expression and molecular mechanism in brain regions differentially affected by AD would represent a significant advance in the genetics of AD.

Most of the current genome-wide studies for AD pathogenesis focus on the hippocampus (HIP) since it is the first and most degraded region in AD brain. However, the changes of gene expression profiles, pathways, and regulatory networks are related to many brain regions which have close relationship to human learning and memory. For example, entorhinal cortex (EC) works as a hub in a widespread network for memory and navigation. The EC-hippocampus system plays an important role in declarative and spatial memories. Posterior cingulate cortex (PC) is a polymodal association area that contributes importantly to normal recognition memory and plays a critical role in visual perception. The functions of middle temporal gyrus (MTG) are associated with brain processes like recognizing familiar faces, ascertaining distance, and understanding meaning of words while reading. Superior frontal gyrus (SFG) is involved in self-awareness and in coordination with the action of the sensory system. The visual cortex (VCX) of the brain, which is responsible for processing visual information, has shown many changes in aging and AD.

In the past decades, many efforts have been made to explore the differentially expressed genes of different AD-affected brain regions. For instance, Loring et al. found that 118 significant genes were differentially expressed in the amygdala and cingulate cortex [4]. Dunckley et al. found 225 differentially expressed genes up- or downregulated in the early stages of NFT formation by comparing gene expression profiles of NFT-bearing with non-NFT-bearing entorhinal cortex neurons [5]. Liang et al. provided gene expression profiles of six brain regions from the healthy and AD-affected individuals. And then they identified differential expression changes of genes in AD pathogenesis, particularly with regard to tangle and plaque formation [6].

Recently, fast development of statistically computational tools enables large-scale discovery of coregulated gene groups and reveal of functional subnetworks for AD. Ray et al. identified 6 coexpressed gene modules, each of which represented some biological processes perturbed in the HIP of AD brain [7]. By using a weighted gene coexpression network analysis method, Miller et al. identified 12 distinct modules related to synaptic and metabolic processes and immune response in the HIP of AD patients [8]. Ray and Zhang developed a novel differential topological method to identify the coexpression network of four regions, HIP, EC, PC, and MTG from AD-affected brain, and built the topological overlap between them [9]. Liu et al. discovered the relationships among AD related pathways and dysfunctions in the six brain regions and identified the similarities and differences of these dysfunctional pathways by integrating protein-protein interaction (PPI) data [10, 11]. Liang et al. identified hub genes and the significantly perturbed subnetwork closely related to plaques and tangles from six AD-affected brain regions by using a heaviest induced subgraph algorithm with a modular scoring function [12]. Chen et al. identified gene signatures associated with six different brain regions. Functional analyses revealed that the biological processes involved with metabolism, protein ubiquitination, vasculature, and synaptic signaling pathways were dysregulated and perturbed in AD [13].

In short, the common features of the dysfunctional pathways and subnetworks extracted from various AD brain regions can provide cooperativities for potential pathogenesis. On the other hand, the distinct features and the differences among different brain regions may provide more enlightenment for finding the pathogenesis of AD. In this study, both common coexpression and specific subnetworks of six AD-affected brain regions were extracted and analyzed to find the underlying AD pathogenesis. Considering that the gene expression networks have been constructed with poor precision and accuracy due to the inherent shortcomings of small sample size and strong noise, protein-protein interaction (PPI) data was added to provide abundant translation information for extracting significant genes and functional subnetworks in this research. The heuristic algorithms and node-scoring functions were applied to integrating gene expression profiles with PPI network information to find out not only common coexpression networks, but also specific dysregulated pathways for six AD-affected brain regions, including HIP, EC, PC, MTG, SFG, and VCX. Then, simulated annealing algorithm was applied to building and optimizing the functional modules. The molecular biological analysis revealed that the inflammation reaction and calcium ions metabolism constructed by the common genes of six brain regions play important roles in AD pathogenesis. Moreover, the identified specific subnetworks for each brain region revealed many biological pathways perturbed in AD which will lead to greater insight into AD pathogenesis.

## 2. Methods

### 2.1. Score Function Principle

Score function can be used as a method for measuring the significance of genes and subnetworks. It includes edge score and node score, of which edge score represents the strength of the correlation between two node-genes and the node score represents the differential significance of each individual gene [12, 14]. The node score function can be given as follows:

(1) $$\begin{array}{c} \text{Score}\_n=\log\left(\frac{ax^{a-1}}{a\tau^{a-1}}\right)=\left(a-1\right)\left(\log\left(x\right)-\log\left(\tau \left(\text{FDR}\right)\right)\right), \end{array}$$

where *a* represents the maximum-likelihood estimation of the shape parameter for the beta-uniform mixture (BUM) model, which indicates that the signal component is equal to the *β*(*a*, 1) density, *x* denotes the raw *P* values, and *τ* represents the significance threshold, which controls the false discovery rate (FDR) for the positively scoring *P* values and fine-tunes the discrimination of signal and noise. The raw *P* values, which are considered as a mixture of signal and noise, can be calculated from the raw gene expression data. By this method, the noise of raw *P* values can be easily separated since the signal component is assumed to be beta (*a*, 1) distributed, and the noise is uniform (0,1) distributed [15].

The values of edge score represent the strength significance of the interaction between genes. Positive score represents activation and negative score represents inhibition. The edge significance score is given as follows:

(2) $$\begin{array}{c} \text{Score}\_e=\mathrm{cov}\left(X,Y\right)=\text{corr}\left(X,Y\right)\text{std}\left(X\right)\text{std}\left(Y\right), \end{array}$$

where *X* and *Y* denote two different genes *X* and *Y*, respectively, and corr(*X*, *Y*) denotes the Pearson correlation coefficient of the gene expression profiles of *X* and *Y*. The differential expressions of the genes are measured as the overall expression variation (std(*X*) and std(*Y*)). In order to avoid the influence from the number of edges, the edge score function is defined as follows:

(3) $$\begin{array}{c} \text{Score}\_e\left(G\right)=\frac{\sum_{e\in E}\text{Score}\left(e\right)-{\text{avg}}_{k}}{{\text{std}}_{k}}, \end{array}$$

where avg_*k*_ denotes the mean of the edge score of the network and std_*k*_ represents the standard deviation of edge scores.

### 2.2. The Algorithm of Identifying Differential Significance Subnetworks

The heaviest induced subgraph algorithm (Heinz) based on the node scoring was applied to our study to find out differentially significant genes and optimal subnetworks from PPI data for different brain regions. The theoretical model of Heinz algorithm belongs to a Steiner-tree problem. The main task of the model is to find an optimal network from a very complex network. In this paper, relevant subnetworks with maximal score are captured from the PPI network with negative and positive scores.

The steps of identifying a significant subnetwork by Heinz algorithm are as follows: firstly, calculate the scores of all the nodes by the score function. Next, define the edge scores based on the node scores connected to the edge. Based on these edge scores, a minimum spanning tree (MST) was calculated. Then, identify all the paths between positive nodes and at the same time the negative nodes involved in these paths were caught. Finally, calculate MST again based on the negative nodes from the obtained maximal significance subnetwork; then, the maximal subnetwork can be finally identified according to the scores of the final positive and negative nodes.

In order to increase the accuracy of the significance subnetwork, in our study, simulated annealing algorithm based on edge scores was applied to removing the weak interactions and enhancing the strong interactions of the calculated significance subnetwork. Guo et al. applied this method to analyzing human prostate cancer and yeast cell cycle. Their results demonstrated that the edge-based method was able to efficiently capture relevant protein interaction behaviors under the investigated conditions [14]. Simulated annealing algorithm is a widely used intelligent optimization algorithm in a number of fields [16]. The modular analysis of biological networks in the bioinformatics research can be considered as a large-scale combinatorial optimization problem essentially. Meanwhile the simulated annealing algorithm is an effective approximation algorithm for solving these kinds of large-scale combinatorial optimization problems with the advantage of avoiding falling into the local optimization.

## 3. Results and Discussion

### 3.1. Data and Preprocessing

The gene expression datasets of healthy elders and AD patients we used in this study were downloaded from NCBI GEO Datasets-record of GSE5281. The neurons were collected by laser-capture microdissection from six different brain regions, including HIP, EC, MTG, PC, SFG, and primary visual cortex (VCX). The human GeneChips Affymetrix U133 Plus 2.0 array was used to provide the gene expression data. Each gene chip involved 54675 genes probes for each sample. The datasets consisted of 13 control (normal aging) and 10 AD-affected samples for HIP, the same sample number for EC, 12 control and 16 AD-affected samples for MTG, 13 control and 9 AD-affected samples for PC, 11 control and 23 AD-affected samples for SFG, and 12 control and 19 AD-affected samples for VCX. Moreover, the PPI datasets we utilized in this research are obtained from the Human Protein Reference Database (HPRD) [17], which consisted of 36504 interactions among 9386 genes.

Before searching for differential significance subnetworks with maximal scores, we matched the preprocessed gene expression data with PPI dataset to get the raw interactions of genes (nodes) with the related edges, and the raw *P* values of all the nodes were calculated as well. Secondly, we processed the gene expression data by gene annotation and variance analysis. For PPI dataset, self-loops and proteins without expression values were removed for simplifying the raw protein interaction networks. Next, the Affymetrix probe set IDs and HPRD gene symbols were mapped to Entrez Gene IDs to extract maximal network. After the preprocessing, there are around 6100–6400 genes left for each brain region.

### 3.2. Results and Discussion

According to our experiments, adjusting the FDR into different values will obtain different number of genes with positive scores; in order to insure plenty of gene nodes with positive scores, for each brain region, we selected different suitable FDR for the PPI network by which each raw network can contain about 15% positive score nodes. The FDR for HIP, EC, MTG, PC, SFG, and VCX region were set to be 0.008, 0.004, 0.0007, 0.01, 0.06, and 0.09, respectively. The node scores and edge scores for each brain region dataset were calculated. Starting from the positive score nodes, Heinz algorithm was applied to searching for the maximal scoring subnetworks in each brain region. After that the simulated annealing algorithm was applied to optimizing the networks with the threshold value of 0.8. By using the simulated annealing algorithm, the interactions with the edge strength exceeding the threshold were added to the extracted subnetworks, while the weak strengths whose value was less than the threshold were removed from the subnetworks. Six differential significance subnetworks were finally identified for the six brain regions, respectively. Based on that, we carried out the functional enrichment analysis for the identified subnetworks by Gene Ontology (GO) and DAVID [18]. Many known risk genes and pathways were extracted in our results such as APP and GAPDH. Additionally, NF-*κ*B signaling pathway, pathways associated with mitochondria, nerve tissue, calcium ion metabolism, and process of acetylation were also identified and shown to be closely associated with the pathogenetic mechanism of AD.

**Figure 1:**
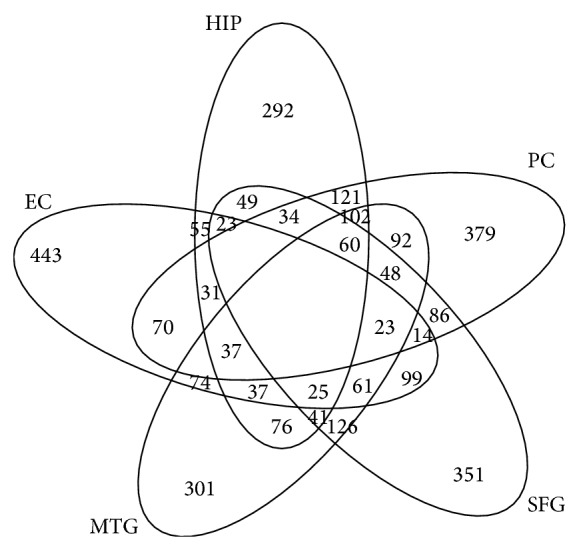
The Venn diagram of five brain regions with significant genes.

By observing the genes and interactions of these six identified significance subnetworks, it was found that they were overlapped with each other. The overlapped genes and interactions may suggest that the similarities may play important roles in the dysregulated networks in AD.  Figure 1 provided the Venn diagram of the overlap of the significance subnetworks among five brain regions.

From Figure 1 we can see that many significantly expressed genes overlapped between different brain regions. Additionally, many other genes were specifically differentially expressed in each brain region as well. Therefore, the consideration for both common and specific genes and subnetworks were necessary to discover the pathogenetic mechanism of AD.

#### 3.2.1. Molecular Biological Analysis of Common Functional Subnetworks

**Figure 2:**
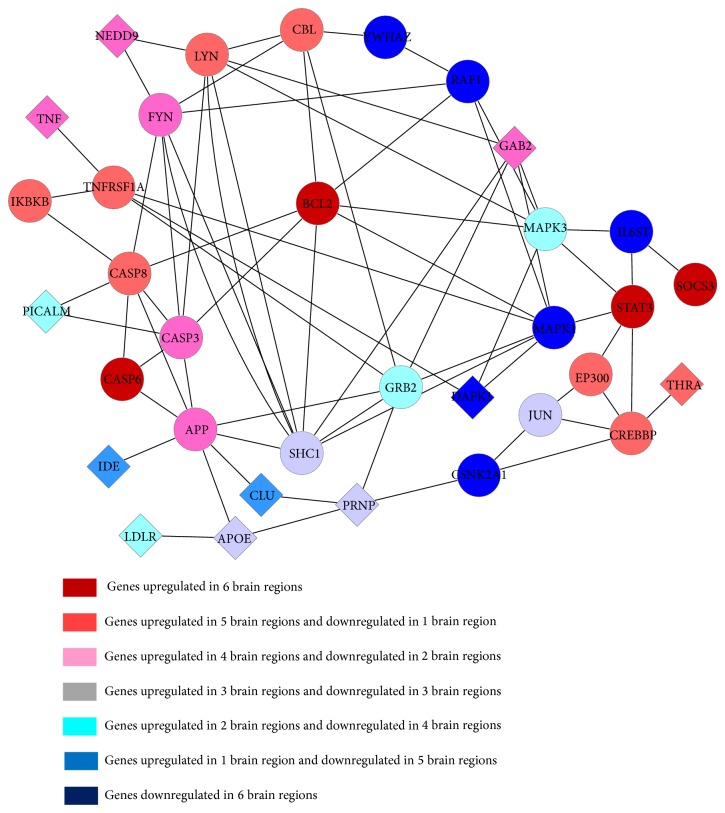
Functional subnetwork of the inflammatory response constructed by common genes.

For the genes overlapped in overall the six brain regions, we selected a gene as a common gene when the number of interactions with other genes exceeds 90% quantile in different subnetworks. With this criterion, 206 common genes were extracted. It showed that most of the common genes play important roles in different brain regions. The molecular biological analysis revealed that many common genes were functionally related to metabolism, synaptic vesicle-mediated transport, transcriptional regulation, protein kinase phosphorylation, apoptosis, intracellular signaling, and cell cycle. Particularly, two functional subnetworks consisted of the common genes, which closely related to inflammation and calcium imbalance, were found distinctly dysregulated in all of the AD-affected brain regions.  Figure 2 showed the inflammatory response subnetwork constructed by the related common genes. Diamonds in Figure 2 denoted the known risk genes of AD, circles presented our extracted common genes related to inflammation, and their different colors indicated different numbers of brain regions in which the gene was upregulated or downregulated.  Table 1 provided the KEGG pathway analysis of genes in Figure 2.

From Figure 2 and Table 1 we can see that many regions of the AD brain suffered from inflammation. The degeneration of tissue and the deposition of beta-amyloid (A*β*) and poly-Tau protein are known as the classical stimulants of inflammation [19]. Mitogen-activated protein kinases (MAPKs) are serine-threonine kinases that mediate various types of cellular activities including cell proliferation, differentiation, survival, death, and transformation [20, 21]. The dysregulation of MAPK signaling pathways was found to have involved in many human diseases including AD, Parkinson's disease (PD), amyotrophic lateral sclerosis (ALS), and many kinds of cancers [22]. The activation of ERK, JNK, and p38 signaling pathways may lead to neuronal apoptosis in AD [23]. In Figure 2, our results indicated that the crowd MAPK1 and MAKP3 expressions were lower than the normal samples obviously in most of the six AD brain regions.

Our results in Figure 2 also showed that caspase-3, Bcl2, caspase-6, and caspase-8 were overexpressed in most of the six AD brain regions. Caspases are a family of cysteine proteases that plays an important role in apoptosis (programmed cell death), necrosis, and inflammation [24, 25]. The predominant caspase involved in the cleavage of amyloid-beta precursor protein (APP) is suggested to be associated with neuronal death in AD [26]. The Bax gene was the first identified proapoptotic member of the Bcl2 protein family; Bax and caspase-3 are both death effectors in neurodegenerative pathways [27]. It implicates that nervous cells apoptosis will occur. The extracted common genes in our results are in keeping with recently pathological analysis. Furthermore, our results also showed that APP was overexpressed in AD samples in all of the six brain regions. The overexpression of APP would activate caspase-3 in human postmitotic neurons and cause the degeneration of postmitotic neurons in AD [28].

The SOCS (suppressors of cytokine signaling) family of proteins has a dual identity; they are the inhibitors of JAK (Janus kinase) signal transducer and the activator of the STAT signaling pathways as well [29]. The subtype SOCS3 has an important function in inhibiting some inflammatory genes, cytokine signaling, and STAT3 activation. The STAT3 has dual function in different types of cells; on the one hand, it promotes proliferation and prevents apoptosis; on the other hand, it can induce growth arrest and apoptosis [30, 31]. Our data exhibited that SOCS3 and STAT3 were both overexpressed in six AD brain regions.

Furthermore, TNFRSF1A protein works as a regulator of inflammation, and as a receptor of TNF-*α* (tumor necrosis factor-alpha) it can activate NF-*κ*B (nuclear factor kappa-light-chain-enhancer of activated B cells) and mediate apoptosis [32]. Our data show that TNFRSF1A and TNF-*α* were both overexpressed. The results confirmed that the six AD brain regions closely associated with inflammation and apoptosis.

**Figure 3:**
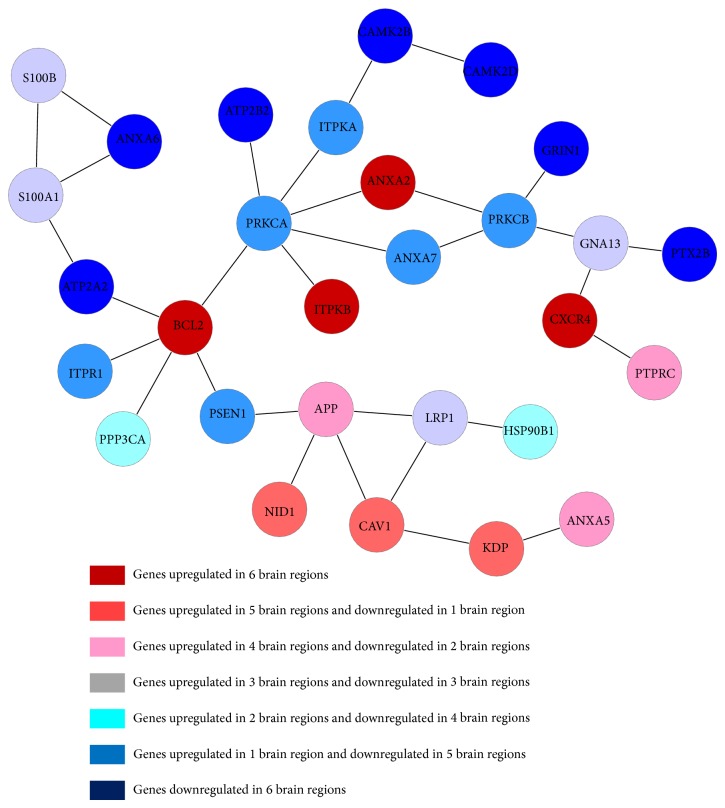
Functional subnetwork of the calcium ion mechanism constructed by common genes.

In addition to inflammatory response, another important subnetwork we found from the common genes was calcium ion metabolism subnetwork.  Figure 3 showed the subnetwork of the calcium ion mechanism with the extracted common genes, and the KEGG pathway analysis of this subnetwork was provided in Table 2.

In AD-affected brain regions, the calcium ion metabolism related signaling were found dysregulated. It was reported that calcium can modulate many neural processes, including synaptic plasticity and apoptosis. With the increase of the intracellular calcium, the accumulation of amyloid-*β*, the hyperphosphorylation of Tau, and neuronal death will occur in the affected brain regions [33]. Particularly, in this subnetwork, our results showed that APP was greatly upregulated in HIP, PC, MTG, and VCX. In addition, Annexin is a Ca (2+)-effector protein which plays an important role in the metabolism of intracellular Ca (2+) [34]. Annexin A2 is a calcium-dependent phospholipid-binding protein whose function is to help organize exocytosis of intracellular proteins to the extracellular domain.  Figure 3 showed that ATP2A2 and ATP2B2 were lower expressed than normal samples in overall the six AD brain regions. ATP2A2 and ATP2B2 are enzymes that can remove bivalent calcium ions from eukaryotic cells against very large concentration gradients and play a critical role in intracellular calcium homeostasis [35, 36].

#### 3.2.2. Specific Subnetworks in Each Brain Region

**Figure 4:**
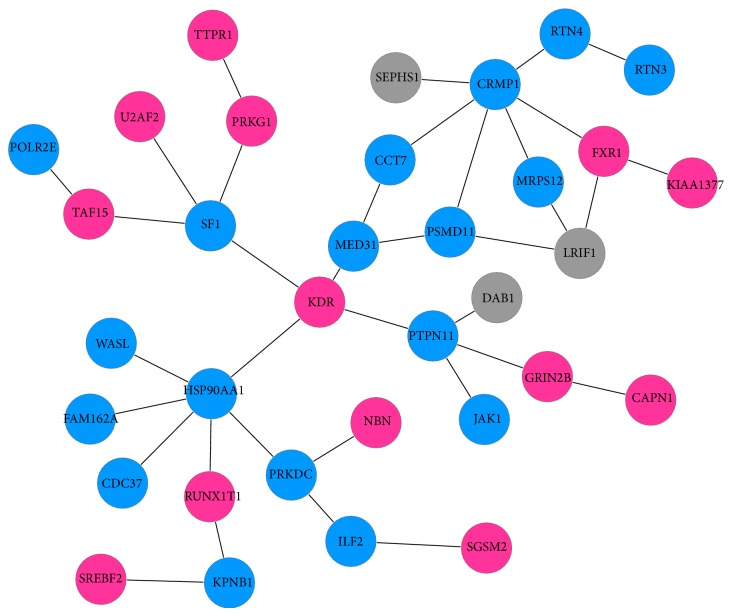
Specific subnetwork of HIP.

**Figure 5:**
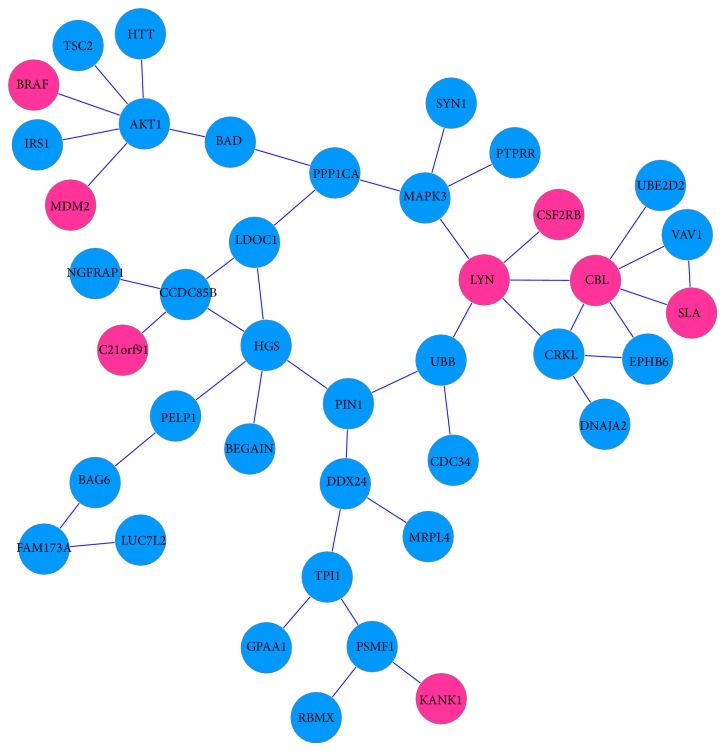
Specific subnetwork of EC.

**Figure 6:**
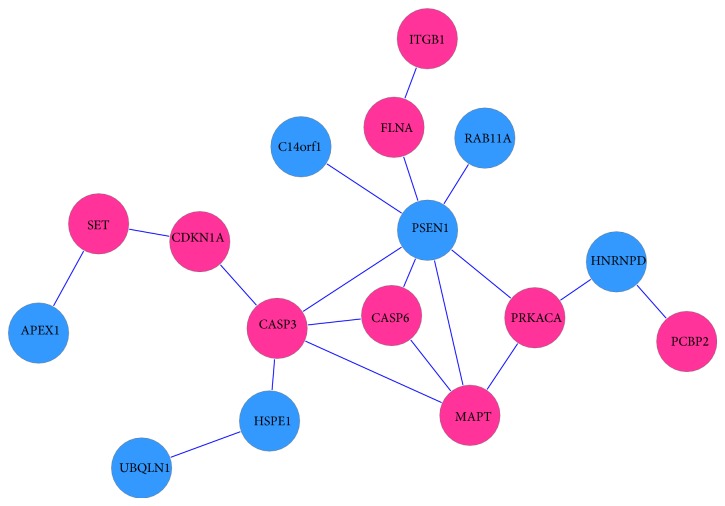
Specific subnetwork of PC.

**Figure 7:**
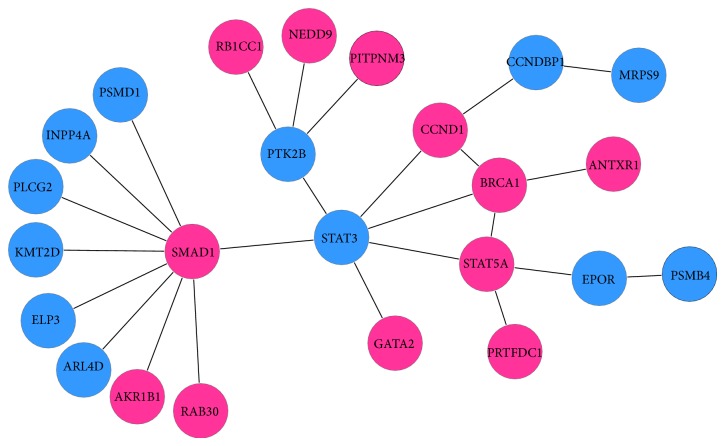
Specific subnetwork of MTG.

**Figure 8:**
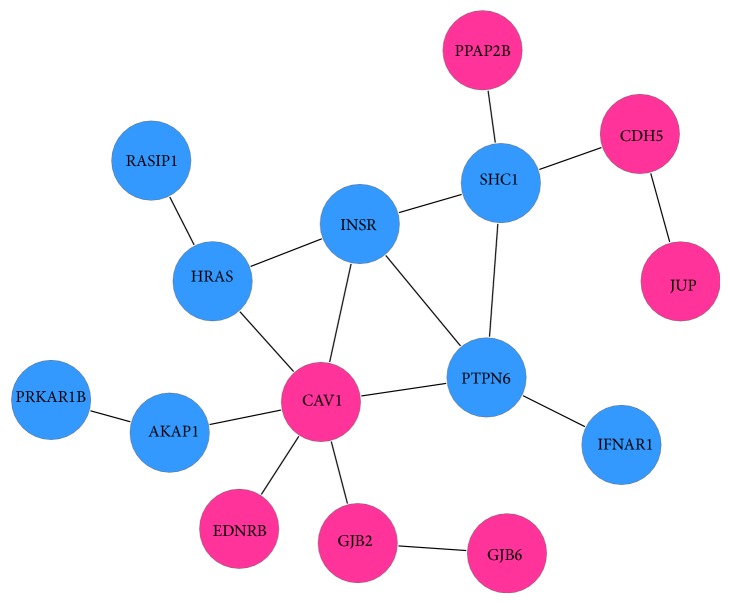
Specific subnetwork of SFG.

Figure 1 revealed that many significant genes were specially expressed in different brain regions. It suggested that some specific dysregulated pathways and subnetworks among them will provide deep insights into the pathogenetic mechanism of AD. By getting rid of the common genes overlapped in different brain regions, the maximal scoring function and the simulated annealing method were used again to construct the specific functional subnetwork by the specifically differential significant genes for each brain region including HIP, EC, PC, MTG, and SFG. Therefore, the sizes of the constructed specific subnetworks are much smaller. Since there were not enough significant genes that can be discovered to construct any functional subnetwork, the result of primary visual cortex (VCX) was absence. Figures 4–8 showed the specific functional subnetworks in HIP, EC, PC, MTG, and SFG, respectively. In Figures 4–8, red circles represented the genes upregulated in this brain region for AD samples, blue circles denoted the genes downregulated, and grey ones denoted that this gene had no great changes compared with normal samples in this brain area.

For HIP, among the specific significant genes, 33 genes can be constructed to the maximal scoring subnetwork (Figure 4). It is known that for AD patients the hippocampus is one of the first regions of the brain to suffer damage including memory loss and disorientation. In this region, some genes were specially differentially overexpressed, such as CAPN1, FXR1, GRIN2B, ITPR1, KDR, KIAA1377, NBN, PRKG1, RUNX1T1, SGSM2, SREBF2, TAF15, and U2AF2. CAPN1 (calcium-activated neutral protease) is a kind of nonlysosomal intracellular cysteine protease. The overexpression of CAPN1 has a relationship with intractable epilepsy as well as the clinicopathological characteristics in AD patients [37]. It was interesting to note that kinase 1 was low expressed in AD, but it was known to be high expressed in cancer.

In EC area, 40 specially expressed genes were constructed to the maximal scoring subnetwork (Figure 5). The changes in the Tau protein and the cleaved fragments of APP have been found in EC region in the early stages of AD [38]. In this brain area, our data showed that BRAF, C21orf91, CBL, CSF2RB, LYN, MDM2, SLA, and KANK1 were all overexpressed. BRAF, as a member of the RAF kinase family of growth signal transduction protein kinases, can affect cell division, differentiation, and secretion by regulating the MAP kinase/ERKs signaling pathway [39]. Members of the Casitas B-lineage lymphoma (Cbl) protein family are evolutionarily conserved multidomain regulators of signal transduction. Colony stimulating factor 2 receptor *β* (CSF2RB) is a risk factor in both schizophrenia and major depression since the overexpression of it has relationship with the disturbance of nerve signal conduction [40]. AKT1 is a survival factor and the activated AKT1 plays important roles in inhibiting apoptosis and promoting cell survival [41, 42]. Our results showed that AKT1 was low expressed in EC of AD patients which indicated that apoptosis was happening.

Figure 6 shows the maximal scoring subnetwork of PC area constructed by 16 specifically significant genes. PC is a polymodal association area that contributes importantly to normal recognition memory and plays a critical role in visual perception [43, 44]. From Figure 6 we can see that genes CASP3, CASP6, CDKN1A, FLNA, ITGB1, MAPT, PCBP2, PRKACA, and SET were overexpressed in PC area of AD brain. The genes CASP3 and CASP6 are related to apoptosis. CDKN1A (p21) has a function of regulating cell cycle by inhibiting the activity of cyclin-CDK2, cyclin-CDK1, and cyclin-CDK4/6 complexes and it activates CDK2; thus, it leads to apoptosis [45, 46]. The Tau proteins are the product of alternative splicing from a single gene that is designated MAPT (microtubule-associated protein tau) in humans, and the overexpression of Tau will impact a neuroinflammation gene expression network perturbed in AD [2, 47, 48]. The major human AP endonuclease APE1 are reported to play an important role in the base excision repair (BER) pathway [49]; however, they were found to be low expressed in PC area of AD brains.

In MTG area, 24 specifically significant genes were extracted to achieve a maximal scoring subnetwork (Figure 7). The functions of MTG are associated with brain processes like recognizing familiar faces, ascertaining distance, and understanding meaning of words while reading. Our data exhibited that ANTXR1, BRCA1, CCND1, GATA2, KMT2D, NEDD9, PITPNM3, PLCG2, PRTFDC1, RB1CC1, SMAD1, and STAT5A were overexpressed. The ANTXR1 is a member of the aldo/keto reductase superfamily, which consists of more than 40 known enzymes and proteins. Aldose reductase contributes to diabetes-mediated mitochondrial dysfunction and damage through the activation of p53. The degree of mitochondrial dysfunction and damage determines whether hyperactivity (mild damage) or apoptosis (severe damage) will ensue [50]. BRCA1 is part of a complex that repairs double-strand breaks in DNA [51]; the overexpression of BRCA1 may suggest that DNA damage is serious; but BRCA1 mutation carriers are at an increased risk of prostate and breast cancer [52]. CCNDBP1 belongs to cyclin D family. The expression of cyclin D suggests that act to link growth factor signals with cell cycle transitions during G1 [53].

For the SFG area, there were 15 specifically significant genes that can be used to construct a maximal scoring subnetwork (Figure 8). SFG is involved in self-awareness and in coordination with the action of the sensory system [54]. In this region, genes CAV1, CDH5, EDNRB, GJB2, JUP, GJB6, and PPAP2B were found overexpressed in AD brains. The CDH5 gene is a classical cadherin from the cadherin superfamily, which provides a molecular system reflecting both early embryonic and mature nervous system architecture. In AD crowd, overexpression of cadherins may be related to restoration of neural epithelium [55, 56]. The PTPN6, which is a member of the PTP (protein tyrosine phosphatase) family, was downregulated in SFG area of AD brain. PTP family is reported to have the ability to regulate a variety of cellular processes including cell growth, differentiation, mitotic cycle, and oncogenic transformation [57, 58].

## 4. Conclusions

AD progression is known to occur in many brain regions which have close relationship of human learning and memory with particular features. Discovering the common and specific changes and dysregulated pathways of different brain regions will provide deep insights for finding of the pathogenesis of AD. In this study, we applied a method of scoring function and simulated annealing algorithm to constructing and optimizing the differential significance subnetworks for six brain regions including hippocampus (HIP), entorhinal cortex (EC), middle temporal gyrus (MTG), posterior cingulate cortex (PC), superior frontal gyrus (SFG), and primary visual cortex (VCX). The common genes we identified from overall the six brain regions revealed two significant functional subnetworks which showed that the dysregulation of inflammation and calcium metabolism play important roles in the onset and deterioration of AD. For example, the dysregulated MAPK signaling pathways and JNK or p38 signaling pathways, as parts of inflammation subnetwork, were demonstrated to be associated with many cellular activities and neuronal apoptosis in AD. Many common genes such as caspases, SOCS3, STAT3, TNFRSF1A, and TNF-*α* were confirmed to have close association with inflammation and apoptosis in all the six AD brain regions. In calcium ion mechanism subnetwork, many genes such as ATP2A2 and ATP2B2 played a critical role in intracellular calcium homeostasis. The dysregulation of intracellular calcium would lead to the accumulation of amyloid-*β*, hyperphosphorylation of Tau, and neuronal death which are parts of the known pathogenesis of AD.

Although we highlighted the contributions of the inflammation and calcium imbalance subnetworks as common features for six brain regions, this paper illustrated the specific dysregulated subnetworks in each AD-affected brain region for HIP, EC, PC, MTG, and SFG. In the subnetwork of HIP, many differentially expressed genes were identified as the clinicopathological characteristics of AD. EC, as another area early affected by AD, was characterized by changes in the Tau protein and APP. Many significant genes in EC played central roles in regulating the MAP kinase/ERKs signaling pathway and affected cell division, differentiation, and secretion. The specific expressed genes in PC area showed close relationship of apoptosis and cell cycle progression. Particularly, the overexpression of Tau will impact a neuroinflammation gene expression network perturbed in AD. The specifically expressed genes in the subnetwork in MTG area of AD showed that they effected the mitochondrial dysfunction and DNA damage. The significant genes related to cadherins in SFG area suggested that this area may contribute to the formation and maintenance of segmental and functional nervous system structures.

In summary, our molecular biological analysis demonstrated that the identified common and specific maximal scoring subnetworks help in comparing biological phenomena across AD-affected brain regions and obtaining a global overview of the disease, which can enable us to further understand the pathogenetic mechanism of AD.

## Figures and Tables

**Table 1:** KEGG pathway analysis results.

Pathway	Number of genes
Pathways in cancer	11
Renal cell carcinoma	6
Neurotrophin signaling pathway	7
Chronic myeloid leukemia	6
Fc epsilon RI signaling pathway	6
Focal adhesion	8
ErbB signaling pathway	6
Prostate cancer	6
Jak-STAT signaling pathway	7
T cell receptor signaling pathway	6
Chemokine signaling pathway	7
Insulin signaling pathway	6
Fc gamma R-mediated phagocytosis	4
GnRH signaling pathway	4
Melanogenesis	4
Toll-like receptor signaling pathway	4
Endometrial cancer	3
Nonsmall cell lung cancer	3
Wnt signaling pathway	4
NOD-like receptor signaling pathway	3
Epithelial cell signaling in *Helicobacter pylori* infection	3
Long-term depression	3
Pancreatic cancer	3
B cell receptor signaling pathway	5
Adherens junction	5
Prion diseases	4
Colorectal cancer	5
Apoptosis	5
Acute myeloid leukemia	4
Glioma	4
Natural killer cell mediated cytotoxicity	5
Long-term potentiation	4
Alzheimer's disease	5
MAPK signaling pathway	6
TGF-beta signaling pathway	3
Gap junction	3

**Table 2:** KEGG pathway analysis results.

Pathway	Number of genes
ErbB signaling pathway	7
Gap junction	7
Calcium signaling pathway	8
Focal adhesion	8
Glioma	6
GnRH signaling pathway	6
Nonsmall cell lung cancer	5
Natural killer cell mediated cytotoxicity	5
Tight junction	5
*Vibrio cholerae *infection	4
Chemokine signaling pathway	5
Pathways in cancer	6
Melanogenesis	4
Leukocyte transendothelial migration	4
Neurotrophin signaling pathway	4
MAPK signaling pathway	5
Insulin signaling pathway	4
Wnt signaling pathway	4
Endometrial cancer	3
Epithelial cell signaling in *Helicobacter pylori* infection	3
Phosphatidylinositol signaling system	4
VEGF signaling pathway	4
Fc epsilon RI signaling pathway	4
Long-term potentiation	3
Adherens junction	3
Colorectal cancer	3
Prostate cancer	3
Fc gamma R-mediated phagocytosis	3
Vascular smooth muscle contraction	3
Dorsoventral axis formation	2
